# Retrospective Analysis of Inflammatory Markers and Patient Characteristics in Hospitalized Covid-19 Patients: An Early Experience in Louisiana

**DOI:** 10.7759/cureus.10257

**Published:** 2020-09-05

**Authors:** Jennifer L Miatech, Christopher P Yaslik, Hailey E Tarleton, Dylan West, William Kellum, Melanie McKnight, M. Patrick Stagg

**Affiliations:** 1 Internal Medicine Residency Program, Baton Rouge General Medical Center, Baton Rouge, USA

**Keywords:** covid, inflammatory mediators, sars, sars-cov-2 (severe acute respiratory syndrome coronavirus -2), covid 19, sars-cov-2 and covid-19

## Abstract

Background

The community transmission of coronavirus disease 2019 (Covid-19) was detected in Baton Rouge, Louisiana, in March 2020. Several previous studies have reported elevations of inflammatory markers in Covid-19 positive patients and suggested a possible correlation to disease severity.

Methods

We identified 69 patients from Baton Rouge General (BRG) Hospital who were admitted with acute hypoxic respiratory failure and laboratory confirmed positive severe acute respiratory syndrome coronavirus-2 (SARS-CoV-2) between March 13 and April 5, 2020. Demographic and laboratory data were obtained through a review of medical records. Statistical analysis was performed on several inflammatory markers in association with clinical disease severity.

Results

We identified 69 patients with confirmed Covid-19 infection. The mean (±SD) age of the patients was 65±14 years, 68% were male and 32% were female. A total of 13 patients (19%) were considered to have mild disease, 25 (36%) had moderate disease, and 31 (45%) were considered to have severe disease. A total of nine patients died (13%), 25 (36%) have been discharged from the hospital, 20 (29%) remain in the ICU, and 15 (22%) remain admitted to the hospital at the time of writing. Lymphopenia was common among hospitalized patients (39%) and was found to be statistically more pronounced in patients with severe disease (p<0.05). Inflammatory marker elevations were also seen in several patients, with statistically significant elevations in C-reactive protein (CRP) and lactate dehydrogenase (LDH) (p <0.05). We found no statistically significant associations between ferritin, D-dimer, troponin I, body mass index (BMI), or creatine kinase (CK) with disease severity.

Conclusions

During the first three weeks of the Covid-19 outbreak in Baton Rouge, Louisiana, the most common reason for admission amongst Covid-19 positive patients was acute hypoxic respiratory failure. Previously, several studies have suggested a correlation between elevated inflammatory markers and disease severity. The presence of lymphopenia and elevations of CRP and LDH may be helpful in the risk stratification of these patients. In an effort to guide clinical decision making and provide insight into disease severity, further characterization of Covid-19 infection in hospitalized patients is urgently needed.

## Introduction

Severe acute respiratory syndrome coronavirus-2 (SARS-CoV-2) is the novel coronavirus first detected in Wuhan, China, that causes coronavirus disease 2019 (Covid-19). Since the initial detection of the virus, more than 1,133,758 cases have been confirmed worldwide with 13,010 cases confirmed in Louisiana as of April 5, 2020 [1]. The number of cases in Louisiana continues to rise at a dramatic rate. There have currently been 477 reported deaths and 1,803 hospitalizations, with 561 patients requiring mechanical ventilatory support [2]. Recent calculations of data obtained by John Hopkins University suggests that Louisiana ranks amongst the top in the country for deaths per capita [3]. Louisiana is currently considered one of the hotspots of infection in the United States.

In an effort to guide clinical decision making and the appropriate allocation of resources, further characterization of Covid-19 infection in hospitalized patients is urgently needed. A total of 69 patients were retrospectively reviewed with analysis of patient characteristics and laboratory findings including troponin I, absolute lymphocyte count, and several inflammatory markers including lactate dehydrogenase (LDH), creatine kinase (CK), C-reactive protein (CRP), ferritin, and D-dimer. Elevations of several inflammatory markers have been suggested to correlate with disease severity; although, the prognostic value of these tests have yet to be defined. Our study found statistically significant elevations in the inflammatory markers CRP and LDH in patients with severe disease (p <0.05). Lymphopenia was also found to be statistically more pronounced in patients with severe disease (p<0.05).

The aim of this interim report is to describe the demographic characteristics and the analysis of laboratory findings among hospitalized patients with Covid-19 infection in Baton Rouge, Louisiana, in an attempt to provide clinical information and help further characterize disease severity.

## Materials and methods

Study population, setting, and data collection

We included patients with laboratory-confirmed Covid-19 infection who were admitted to Baton Rouge General (BRG) Hospital in Baton Rouge, Louisiana, between March 13 and April 5, 2020. A confirmed case of Covid-19 was defined by positive result on reverse-transcriptase-polymerase-chain-reaction (RT-PCR) assay of a specimen collected on a nasopharyngeal swab. Only laboratory confirmed cases were included, with inconclusive results excluded.

A total of 70 adults (18 years of age or older) were identified from BRG Hospital. Pregnant women, prisoners, and those younger than 18 years of age were excluded from the study, only one pregnant patient was excluded from this study. The BRG institutional review board approved the study with the reliance agreement (also known as an authorization agreement). Informed consent was waived, and researchers analyzed only the identified (anonymized) data without patient identifiers released.

Data was obtained from the search of BRGs electronic medical record for Covid-19 positive patients. We obtained demographic and laboratory data on all hospitalized Covid-19 confirmed patients. Admission data was included for the patient's body mass index (BMI), LDH, CK, CRP, ferritin, D-dimer, troponin I, and absolute lymphocyte counts. All laboratory tests were performed at the discretion of the treating physician.

Study definitions

Coexisting conditions were ascertained from physician documentation. Patient data were censored at the time of data cut off, which occurred on April 5, 2020. For the purpose of this study, disease severity was defined as mild, moderate, and severe disease. Mild disease was defined as patients maintaining oxygen saturation (SpO2) >90% on room air throughout hospitalization. Moderate disease was defined as patients who required supplemental oxygenation via nasal cannula up to 6 L in order to maintain SpO2 >90%. Severe disease was defined as patients who required greater than 6 L of supplemental oxygen to maintain SpO2 >90%. The severe group included patients requiring non-rebreather, high-flow nasal cannula (HFNC), non-invasive positive pressure ventilation (NIPPV), and intubation with mechanical ventilation. Patients who have remained hospitalized at the time of data censoring demonstrated no increases in oxygen requirements for at least three hospital days.

Specimen collection and testing

Clinical specimens for Covid-19 diagnostic testing were obtained in accordance with Centers for Disease Control and Prevention (CDC) guidelines. Laboratory confirmation of SARS-COV-2 was performed at Louisiana State University (LSU) River Road Testing Laboratory in Baton Rouge, Louisiana. RT-PCR assays were performed in accordance with the protocol established by the CDC. Details regarding laboratory confirmation processes are provided in the Supplementary Appendix.

Statistical analysis

Mean [standard deviation (SD)] and ranges were reported for normally distributed, continuous variables. Frequencies and percentages were reported for categorical variables. Statistical analysis was performed using single-variable analysis of variance (ANOVA) to determine statistical significance. Post-hoc analysis was performed using the Bonferroni method. Tests of homogeneity of variances were performed using Levene’s test. Absolute lymphocyte count violated the test of homogeneity prompting further analysis using Welch’s test. All statistical tests were two-tailed, and a p value less than 0.05 was considered statistically significant. No imputation was made for missing data. Analysis was performed with IBM Statistical Package for the Social Sciences (SPSS) software, v26 (SPSS Inc., Chicago, IL).

## Results

Demographic characteristics of the patients

During the period from March 13 through April 5, 2020, we identified 69 patients admitted to the hospital or ICU with confirmed Covid-19 infection at BRG. The demographic characteristics of the patients are shown in Table 1. The mean (±SD) age of the patients was 65±14 years (range 30 to 94); 68% were male and 32% were female; 61% were African-American and 36% were Caucasian. The average BMI was 33.1±8.7. A total of 58% of our patients were considered to be obese with a BMI of ≥ 30.0. Chronic medical conditions were common in all hospitalized patients, including diabetes mellitus (39%), chronic kidney disease or end-stage renal disease (14%), asthma or chronic obstructive pulmonary disease (10%), hypertension (83%), coronary artery disease (12%), and current or former smoking history (26%). A total of 45 patients (65%) had more than one coexisting condition. Individual participant data is provided in the Supplementary Appendix.

**Table 1:** Clinical Baseline Patient Characteristics ◊ ◊ The plus-minus values are means ± SD. Percentages may not total 100 because of rounding. ICU denotes intensive care unit. † The body mass index (BMI) is the weight in kilograms divided by the square of the height in meters. Data on BMI were missing for four patients. § Cancer included patients with active cancer.

Characteristic	Patients (N=69)
Mean age (range) — yr	65±14 (30 to 94)
Sex — no. (%)	
Male	47 (68% )
Female	22 (32% )
Race — no. (%)	
African-American	42 (61%)
Asian	1 (1%)
Caucasian	25 (36%)
Hispanic	1 (1%)
BMI †	33.1±8.7
Coexisting condition — no. (%)	
Asthma / Chronic obstructive pulmonary disease	7 (10%)
Cancer §	9 (13%)
Chronic kidney disease / End-stage renal disease	10 (14%)
Coronary artery disease	8 (12%)
Current or former smoking history	18 (26%)
Diabetes mellitus	27 (39%)
Hypertension	57 (83%)
Immunosuppression / Transplant / Biologics	3 (4%)
Obstructive sleep apnea	4 (6%)
Admission status — no. (%)	
Admitted	15 (22%)
Admitted ICU	20 (29%)
Discharged	25 (36%)
Deceased	9 (13%)
Patient disease severity — no. (%)	
Mild disease	13 (19%)
Moderate disease	25 (36%)
Severe disease	31 (45%)
◊ The plus-minus values are means ± SD. Percentages may not total 100 because of rounding. ICU denotes intensive care unit. † The body-mass index is the weight in kilograms divided by the square of the height in meters. Data on body-mass index were missing for 4 patients. § Cancer included patients with active cancer.	

Laboratory findings

**Figure 1:**
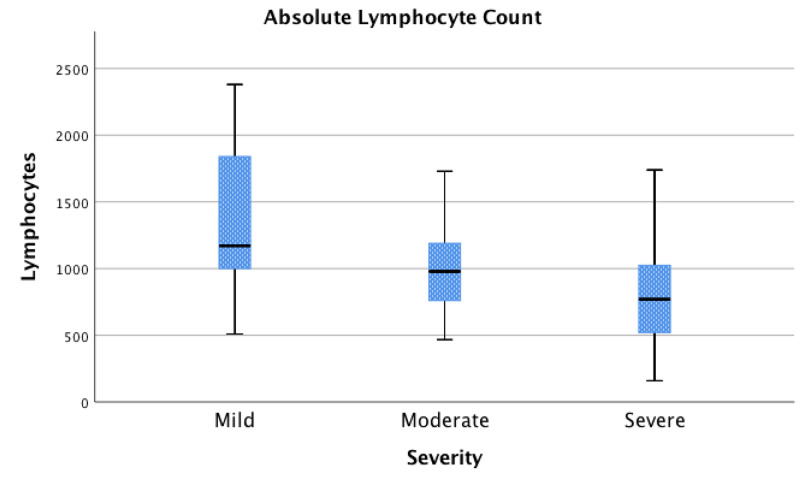
Absolute Lymphocyte Count Association between absolute lymphocyte count and disease severity; interquartile range (IQR) [Q1 - Q3] for mild [1000 – 1840 cells/μL], moderate [760 – 1190 cells/μL], and severe [520 – 1025 cells/μL].

**Figure 2:**
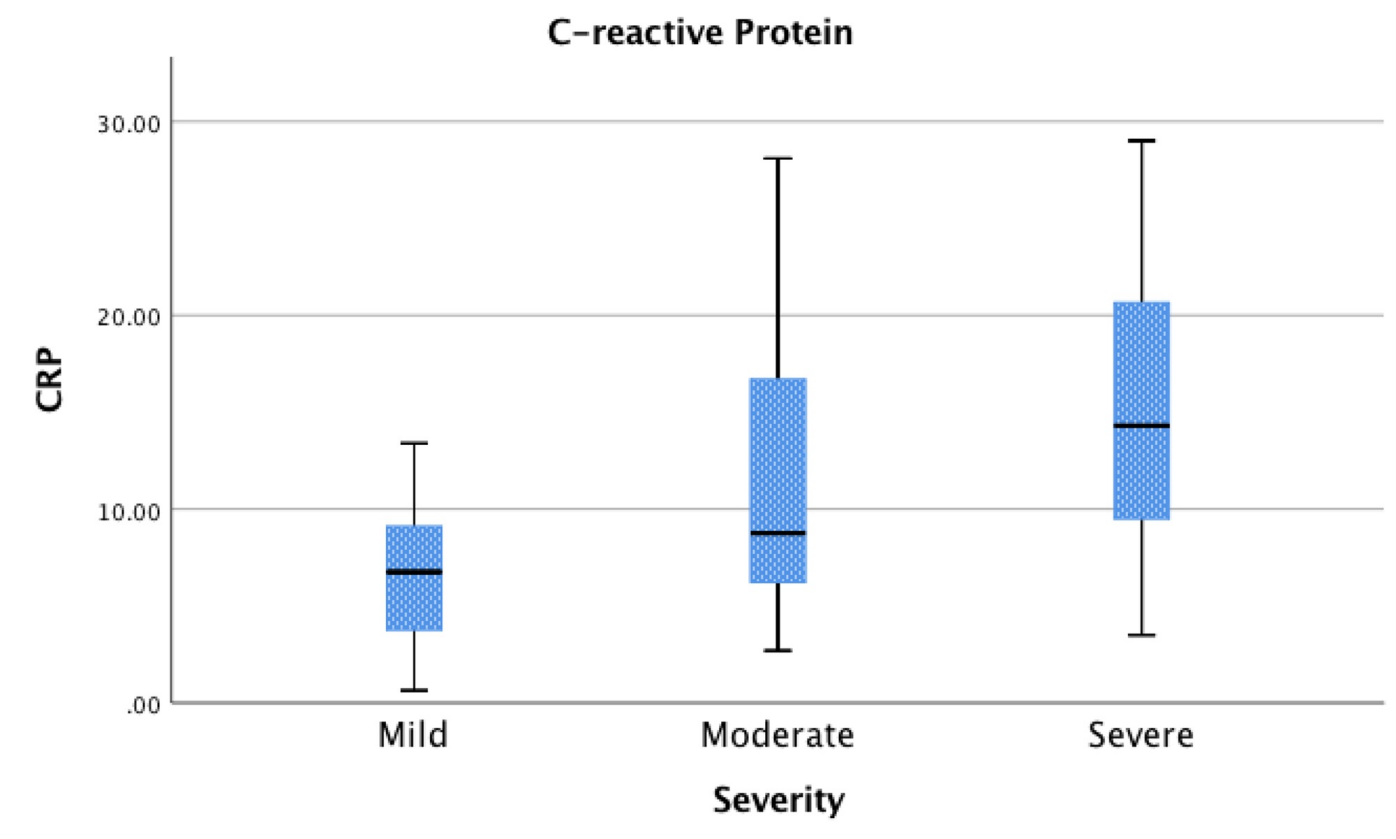
C-Reactive Protein (CRP) Association between C-reactive protein and severity; interquartile range (IQR) [Q1 - Q3] for mild [3.7 – 9.1 mg/dL], moderate [6.2 – 16.7 mg/dL], severe [9.5 – 20.6 mg/dL].

**Figure 3:**
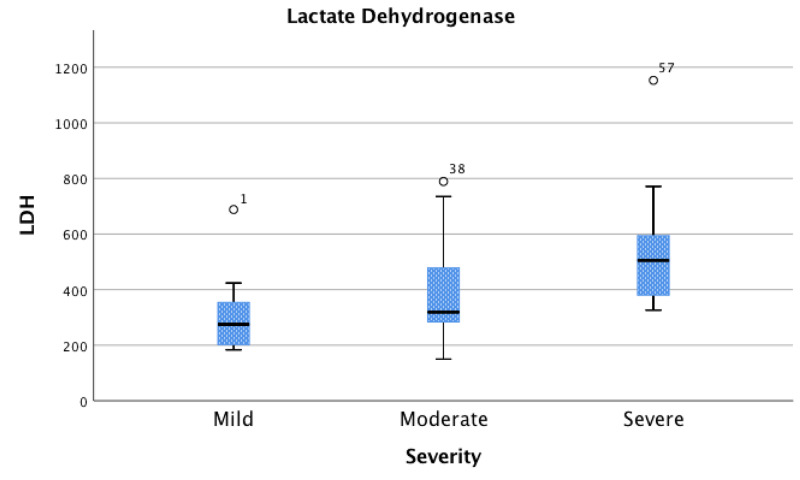
Lactate Dehydrogenase (LDH) Association between LDH and disease severity; interquartile range (IQR) [Q1 - Q3] for mild [202 – 354 U/L], moderate [284 – 478 U/L], and severe [380 – 594 U/L]. The numbers 1, 18, 37 correlate to outliers in the individual participant data table which can be referenced in the Supplementary Appendix.

For the purposes of this study, patients were grouped according to their disease severity based upon oxygen requirements to maintain SpO2 above 90%. A total of 13 patients (19%) were considered to have mild disease, 25 (36%) moderate disease, and 31 (45%) severe disease. Figures 1-3 shows the laboratory findings in patients on admission. Lymphopenia (reference range, 1180-3740 cells/μL) was seen in 51 patients (74%), with a median absolute lymphocyte count of 960. Inflammatory markers were found to be elevated in several patients. CRP was elevated (reference range, 0.001-1.00 mg/dL) in 57 patients (98%); LDH elevated (reference range, 84-246 U/L) in 45 patients (85%); ferritin elevated (reference range, 8-252 ng/mL) in 47 patients (82%); D-dimer elevated (reference range, 0.00-0.39 μg/mL) in 51 patients (91%); troponin I elevated (reference range, 0.000-0.045 ng/mL) in 16 patients (27%); CK elevated (reference rage, 39-308 U/L) in 20 patients (41%).

Association between absolute lymphocyte count, CRP, LDH, and Covid-19 disease severity

Absolute lymphocyte count was reported for all 69 patients (100%) included in this study. Of these, 13 (19%) had mild disease, 25 (36%) had moderate disease, and 31 (45%) had severe disease (Figure 1). A statistically significant association between decreased absolute lymphocyte counts and disease severity was seen between the mild and severe (p=0.000) groups, as well as the mild and moderate (p=0.037) groups. Interpretation of homogeneity of variance using Levene’s test showed significant heterogeneity between groups (p=0.023). Due to this, further investigation using Welch’s test showed statistical significance with a corrected p-value of 0.006.

CRP levels were measured in 58 patients (84%). Of these, 11 (19%) had mild disease, 23 (40%) had moderate disease, and 24 (41%) had severe disease (Figure 2). A statistically significant association between increased CRP levels was seen with disease severity between the mild and severe (p=0.004) groups. No heterogeneity was observed when Levene’s test was performed (p=0.206).

LDH levels were measured in 53 patients (77%). Of these, 11 (21%) had mild disease, 23 (43%) had moderate disease, and 19 (36%) had severe disease (Figure 3). A statistically significant association between higher LDH levels was seen with disease severity between the mild and severe (p=0.040) and the moderate and severe (p=0.007) groups. Levene’s test was performed and no heterogeneity was observed (p=0.794).

Association between ferritin, D-dimer, troponin I, BMI, CK, and Covid-19 disease severity

Ferritin levels were measured in 57 patients (83%). Of these, 11 (19%) had mild disease, 23 (40%) had moderate disease and 23 (40%) had severe disease. We found no association between ferritin and disease severity (p>0.05). A loose association was observed, and additional studies are needed to investigate this further.

D-dimer levels were measured in 56 patients (81%). Of these, 11 (20%) had mild disease, 23 (40%) had moderate disease, and 22 (39%) had severe disease. We found no association between D-dimer and disease severity (p>0.05). Similar to ferritin, a loose association was observed between D-dimer and disease severity.

Analysis of troponin I, BMI, and CK levels revealed no statistically significant association to disease severity (p>0.05). Complete statistical analysis is provided in the Supplementary Appendix.

Outcomes

As of April 5, 2020, of the 69 patients admitted to BRG, nine (13%) died, 25 (36%) have been discharged from the hospital, 20 (29%) remain in the ICU, and 15 (22%) remain admitted to the hospital. A total of 20 patients have required mechanical ventilation. Of these patients, five died, three were extubated, and 12 patients remain on mechanical ventilation. A total of two patients remain on HFNC, two patients on non-rebreather, and two patients on NIPPV. The nine deaths included four patients who had do-not-resuscitate orders in place at the time of hospital admission. African Americans made up a large portion of severe cases, 23 patients (74%), and the majority of deaths, eight patients (89%). 

## Discussion

This single-centered, retrospective, observational analysis describes 69 hospitalized patients who were admitted for acute hypoxic respiratory failure and laboratory-confirmed Covid-19 infection. We included patients with Covid-19 who were admitted at BRG between March 13 and April 5, 2020. Overall approximately half of the patients remain admitted at the time of data censoring, with 29% of patients continuing to require ICU level of care. A total of 25 patients (36%) have been discharged at the time of reporting, a majority of these patients were considered to have mild to moderate disease during hospitalization, with two patients classified as having severe disease, and one requiring mechanical ventilation during admission. The timing of censorship was early on during the pandemic, and therefore, the only treatment practice at the time was supportive care. 

The majority of our patients included in this study were male, with only 32% female. A large proportion of our patients were African American (61%) and Caucasian (36%). African Americans tended to have more severe disease and made up a larger portion of deceased patients. Our admitted patient population with confirmed Covid-19 were older, 65±14 years of age. Among our patient population, obesity was found to be very common within all three disease severity groups. Peng et al. reported that higher BMIs were more often seen in critical patients and non-survivors [4]. Despite this report, no statistically significant differences were found between disease severity and BMI. This finding may be attributed to the prevalence of obesity in our study population, which is estimated to be approximately 33% in Baton Rouge, Louisiana [5]. The majority of patients had coexisting conditions prior to their admission to the hospital, the most common being hypertension and diabetes mellitus. The increased prevalence of multiple comorbidities in our population differs from most studies reported from China; although, the presence of such comorbidities was found to be more common amongst patients with severe disease [6,7].

Lymphopenia has been reported to be a common finding in patients with Covid-19 infection. This finding has been confirmed by several studies and one meta-analysis of patients with confirmed Covid-19 infection [7-11]. In a study of 99 patients, Zhang et al. suggested an association between reduced absolute lymphocyte count and disease severity [12]. Our findings are similar to previous reports of lymphopenia which was present in 74% of our patient population with a statistically significant correlation to severe disease.

Currently, elevation of several inflammatory markers has been suggested to correlate to disease severity; however, the prognostic value of these markers has yet to be established. Massachusetts General Hospital includes several of these markers in their hospital Covid-19 protocol to aid clinicians in assessing disease severity [13]. Zhang et al. reported a strong correlation with elevated CRP, CK, LDH, and D-dimer levels in 95 patients who were considered to have severe disease [12]. A study of 113 deceased patients with Covid found that concentrations of CK, LDH, troponin I, and D-dimer were markedly higher in deceased patients than in recovered patients [10]. Benefits may also be seen when following these inflammatory markers over time. Yuan et al. demonstrated that the COVID-19 messenger ribonucleic acid (mRNA) clearance ratio significantly correlated with the decline of serum CK and LDH levels [14].

In our study, we found statistically significant elevations of only CRP and LDH associated with disease severity but failed to show similar associations with CK or D-dimer. A recent publication in the New England Journal of Medicine by Guan et al. demonstrated trends of lymphopenia and elevations of CRP in 1099 Covid-19 positive patients, but reported less commonly elevated CK and D-dimer levels [7]. In contrast, Zhou et al. found that D-dimer levels greater than 1 μg/mL were associated with higher odds of in-hospital death [11]. Another smaller study also suggested the potential benefit of using inflammatory markers to help with risk stratification. In their study, they found that D-Dimer was closely related to the occurrence of severe Covid-19 in the adult patients, and its combined detection with interleukin-6 (IL-6) had the highest specificity and sensitivity for early prediction of the severity of Covid-19 patients [15]. The differing results may be related to the relatively small sample sizes of some of the studies. Our study failed to demonstrate a correlation with disease severity, particularly with D-dimer and CK levels, which may be attributed to the small sample size. We will continue to check levels of these inflammatory markers for future reporting.

We also investigated the utility of ferritin in the prediction of disease severity. In our study, we did not find a significant association within our patient population. There are only a few reports in the literature of ferritin measurements in patients with Covid-19 infection. These few studies have found increased levels of ferritin in these patients, but no correlation has been made with disease severity [16,17]. One study suggested that the presence of elevated ferritin was more prevalent in severe disease; although, they also reported high values in non-severe patients [11]. We report a similar finding, with 82% of our patients demonstrating elevated ferritin levels.

Troponin I concentrations were elevated in 23% of our patients, with one presenting with ST-elevation myocardial infarction (MI). This patient presented with ST elevations on his electrocardiogram and an elevated troponin I of 5.85 on admission. A case series from China found elevated troponin levels in 28% of their study sample of 187 patients [18]. Lippi et al. reported alternative findings of only marginally increased troponin I levels in only 8-12% of positive cases; although, a more recent meta-analysis of Covid-19 patients performed by the same author suggests that elevations may be seen in more severe cases of disease [19]. This more recent finding was also exemplified in a study of deceased Covid-19 infected patients, which reported higher concentrations of several inflammatory markers, cardiac troponin I, and N-terminal pro-brain natriuretic peptide in these patients [10]. Ruan et al. suggested increased statistically significant mortality in patients with elevated myoglobin and troponin I [20]. Although our study found no significant difference in troponin elevations with disease severity, elevations were seen in 27% of our patients. The cardiac involvement associated with Covid-19 is currently under investigation. 

Our study has several limitations. Notably, several patients had missing laboratory values, and the reports of patient comorbidities relied on electronic medical record documentation. The lack of laboratory data is in part a result of the novelty of this disease entity. Comprehensive testing improved with better clinician understanding and coordination. Another limitation of this study is that 35 patients (51%) have remained hospitalized at the time of data censoring on April 5, 2020. This contributes to the lack of final patient outcomes. Finally, our study has a relatively small sample size compared to larger studies from China due to the fact that Covid-19 was first reported in the United States on January 20, 2020 and the first documented case of Covid-19 at BRG was on March 8, 2020.

Here we report the early experience of Covid-19 at our institution, we expect cases to increase over the coming weeks to months. Currently, few treatment options are available and their potential benefits are still being investigated. These inflammatory markers may aid clinicians in risk stratification to guide clinical decision making. In an effort to guide clinical decision making and provide insight into disease severity, further characterization of the novel Covid-19 disease in hospitalized patients is urgently needed.

## Conclusions

This early experience of Covid-19 in Baton Rouge, Louisiana, reports interim data prior to the arrival of the expected Covid-19 infection peak in one-two weeks. This single-centered, retrospective, observational analysis describes the patient characteristics and laboratory findings of 69 hospitalized patients who were admitted for acute hypoxic respiratory failure and laboratory-confirmed Covid-19 infection. In our study population, African Americans tended to have more severe disease and made up a larger portion of deceased patients. The presence of medical comorbidities were also found to be more common amongst patients with severe disease. Evaluation of inflammatory markers revealed a statistically significant association between lymphopenia, elevated LDH, and CRP with severe disease. This association was not seen with ferritin, D-dimer, troponin I, or CK levels. These associations needs to be investigated further; additional data will be provided in the future following resolution of the pandemic. The utilization of inflammatory markers may have prognostic value to guide risk stratification for potential therapeutic agents. The aim of this preliminary report is to provide further data and understanding of this novel disease.

## Appendices

Supplementary appendix

This appendix has been provided by the authors to give readers additional information about their work.

Supplement to: Miatech JL, Yaslik CP, Tarleton HE, et. al Retrospective Analysis of Inflammatory Markers and Patient Characteristics in Hospitalized Covid-19 Patients - An Early Experience in Louisiana

Table of contents

Methods: Covid-19 Centers for Disease Control Laboratory Testing Protocol: https://www.fda.gov/media/134922/download

Individual research subject data of demographics, comorbidities, laboratory findings, severity, and admission status (Table 2).

**Table 2:** Individual Participant Data

Patient	Age	Sex	Race	Creatine kinase (U/L)	Lactate dehydrogenase (U/L)	C-reactive protein (mg/dL)	Ferritin (ng/mL)	D-dimer (ug/mL)	Troponin I (ng/mL)	Absolute lymphocyte count (cells/uL)	Severity	Status	Body-mass index (kg/m2)	Asthma/ chronic obstructive lung disease	Coronary artery disease	Smoking history	Diabetes mellitus	Chronic kidney disease / end-stage renal disease	Hypertension	Immuno- compromised	Cancer	Obstructive sleep apnea
1	45	Male	African-American	4688	688	10.3	67.1	0.64	0.015	510	Mild	Discharged	32.3									
2	59	Male	African-American	–	224	13.4	1934.1	1.22	–	2380	Mild	Discharged	62.6	X					X			
3	61	Male	Caucasian	490	207	3.1	175.9	0.99	0.015	1170	Mild	Discharged	29.5									
4	51	Female	Caucasian	32	183	2.14	46	0.27	0.015	1150	Mild	Discharged	57.3				X		X		X	
5	54	Female	Caucasian	36	294	7.92	533.2	0.61	0.015	2250	Mild	Discharged	38.9									
6	55	Male	African-American	648	307	6.75	492.1	0.52	0.015	860	Mild	Discharged	45.7		X				X			
7	59	Male	Caucasian	79	424	7.58	410.7	1.19	0.015	570	Mild	Discharged	27.4									
8	87	Female	Caucasian	55	197	0.64	18	0.79	0.015	1840	Mild	Discharged	24.8						X			
9	53	Female	African-American	126	275	5.32	504.5	1.3	0.015	1280	Mild	Discharged	39.3						X		X	
10	30	Male	Caucasian	53	190	4.43	332.8	0.28	0.015	1980	Mild	Discharged	18.8									
11	65	Female	African-American	–	–	–	–	–	0.157	1090	Mild	Discharged	24.9			X			X	X		
12	39	Male	African-American	–	–	–	–	–	–	1730	Mild	Discharged	31.4						X			
13	51	Male	African-American	72	401	11	792.1	0.79	0.015	1000	Mild	Discharged	–				X		X			
14	61	Female	African-American	163	535	28.1	574.9	3.93	0.015	467	Moderate	Discharged	36.7									
15	48	Male	Caucasian	687	347	7.7	837.2	0.5	0.015	1640	Moderate	Admitted	41.5				X		X			
16	83	Male	Caucasian	–	307	6.59	495.6	0.86	0.018	1040	Moderate	Admitted	29.8	X	X	X			X			X
17	55	Male	Caucasian	601	295	19.8	943.8	1.08	0.015	1320	Moderate	Discharged	25.3									
18	66	Male	Caucasian	112	735	8.43	928.1	7.49	0.015	900	Moderate	Admitted	39.3									
19	72	Male	Caucasian	–	–	–	–	–	0.015	750	Moderate	Discharged	30.7	X		X			X			X
20	71	Male	African-American	410	251	17.4	1136.7	1.93	0.162	960	Moderate	Admitted	25.8				X		X		X	
21	73	Female	Caucasian	–	–	–	–	–	–	1560	Moderate	Admitted	21					X	X			X
22	42	Male	African-American	–	300	5.55	1017.5	0.33	–	640	Moderate	Discharged	38.6				X		X			
23	80	Male	Caucasian	70	303	5.93	149.5	1.22	0.038	1200	Moderate	Admitted	40.5	X		X	X		X			
24	59	Male	African-American	–	433	14	726.5	1.6	0.015	1040	Moderate	Discharged	42.8						X			
25	71	Female	African-American	84	288	6.7	123	0.58	0.015	1190	Moderate	Admitted	29.2			X			X			
26	81	Female	Hispanic	440	505	19.6	816.9	3.7	0.324	1320	Moderate	Admitted	–		X		X		X			
27	64	Male	African-American	345	417	11	419.3	0.88	0.015	1010	Moderate	Admitted	51.9			X			X			
28	50	Male	African-American	186	508	16	1310.9	1	0.015	620	Moderate	Discharged	25.7						X			
29	65	Male	African-American	134	239	26.7	365.3	3.55	0.015	670	Moderate	Admitted	34.3	X	X	X	X		X	X		
30	48	Male	African-American	350	478	9.86	345	0.64	0.015	970	Moderate	Discharged	44.8				X		X			
31	70	Female	Caucasian	168	319	8.76	648.5	0.74	0.061	1020	Moderate	Discharged	40.5				X		X			
32	66	Male	Caucasian	312	280	5.17	491.3	0.53	0.535	979	Moderate	Discharged	38.5				X		X			
33	64	Male	Caucasian	18	150	14.3	602.4	0.5	0.015	1090	Moderate	Admitted-ICU	32.4			X	X	X	X		X	
34	70	Female	Caucasian	37	183	2.7	20.3	0.7	0.096	760	Moderate	Admitted	24.1	X		X			X	X		
35	71	Female	African-American	185	392	4.53	229.4	1.18	0.015	890	Moderate	Admitted	32.2				X		X		X	
36	38	Male	African-American	193	249	2.8	363.4	0.27	0.015	1730	Moderate	Admitted	28.5									
37	52	Male	Caucasian	818	789	6.54	831.2	0.27	0.015	650	Moderate	Discharged	32.3						X			
38	62	Female	African-American	151	478	24	1587.8	1.18	0.015	930	Moderate	Admitted	–						X			
49	72	Male	Caucasian	–	–	–	–	–	0.015	510	Severe	Admitted-ICU	31.1				X		X		X	
40	69	Male	African-American	755	387	12.3	811.4	2.44	0.015	1110	Severe	Admitted-ICU	36.8			X			X			
41	56	Female	African-American	446	601	6.99	1003.5	0.93	0.015	1050	Severe	Admitted-ICU	40.3						X			
42	64	Female	African-American	384	511	13.4	484.3	2.38	0.062	810	Severe	Deceased	34.8						X			
43	55	Female	Caucasian	–	–	–	316.7	–	–	1070	Severe	Discharged	35.1				X		X			
44	57	Male	African-American	106	373	4.13	420.6	0.65	0.015	576	Severe	Admitted-ICU	27.2		X	X	X		X			
45	69	Male	Asian	–	–	27.1	–	–	–	170	Severe	Admitted-ICU	25.5									
46	54	Male	African-American	–	–	–	–	–	–	940	Severe	Admitted-ICU	26.7				X		X			
47	76	Male	African-American	–	–	–	–	–	–	365	Severe	Deceased	31.2						X			
48	79	Male	African-American	2379	505	10.7	2750.2	0.82	0.015	1740	Severe	Admitted-ICU	30.1			X		X	X			
49	59	Male	African-American	367	771	5.1	2471.1	3.34	0.015	790	Severe	Admitted-ICU	27.1									
50	75	Female	Caucasian	72	332	8.3	189.9	1.1	0.015	710	Severe	Admitted-ICU	28		X	X	X		X			
51	93	Female	African-American	147	397	29	1230	2.44	0.06	745	Severe	Deceased	–					X				
52	94	Male	African-American	–	588	13.7	739.7	1.24	2.07	1210	Severe	Deceased	24.1						X			
53	86	Male	African-American	–	–	–	–	–	–	420	Severe	Deceased	23.2						X		X	
54	80	Female	Caucasian	477	527	23.8	491	3.7	0.092	1320	Severe	Deceased	30.6						X			
55	83	Male	African-American	487	–	25.3	–	20	5.85	530	Severe	Deceased	22.6		X	X		X	X			
56	71	Male	African-American	197	1153	17.4	17566	1.34	0.029	1165	Severe	Admitted-ICU	36.5				X		X			
57	77	Male	African-American	–	–	12.9	420.4	1.47	0.022	480	Severe	Admitted-ICU	41.2			X		X	X		X	
58	60	Female	African-American	–	–	–	–	–	0.065	630	Severe	Admitted-ICU	31.5						X		X	
59	88	Male	African-American	292	361	7.5	188.2	0.56	0.058	390	Severe	Admitted-ICU	15.4		X		X	X	X			X
60	59	Male	African-American	–	–	14	1588.5	–	0.015	160	Severe	Deceased	29.1						X			
61	65	Male	Caucasian	697	400	17.4	424.1	0.89	0.02	440	Severe	Admitted-ICU	47.4				X		X			
62	81	Male	African-American	–	–	–	–	–	0.115	640	Severe	Deceased	39				X	X	X			
63	82	Male	African-American	139	620	14.6	334.2	20	0.015	1000	Severe	Admitted-ICU	27.1				X		X			
64	49	Male	African-American	–	–	24	792.9	1.22	–	880	Severe	Discharge	25.6			X	X		X			
65	86	Male	Caucasian	295	326	15.4	1013.1	0.48	0.063	770	Severe	Admitted	32.6						X			
66	47	Male	African-American	969	646	17	1974.4	2.14	0.017	760	Severe	Admitted-ICU	40.1			X	X		X			
67	71	Male	African-American	268	329	18.7	7867.2	0.86	0.015	980	Severe	Admitted-ICU	28			X	X		X			
68	76	Female	African-American	122	497	3.5	991.5	3.63	0.076	1320	Severe	Admitted-ICU	29.4				X	X	X			
69	79	Female	Caucasian	122	546	22.6	4738.8	2.88	0.027	950	Severe	Admitted-ICU	37.1	X				X	X			
